# Morphological and Molecular Characterization of Proliferative Inflammatory Atrophy in Canine Prostatic Samples

**DOI:** 10.3390/cancers13081887

**Published:** 2021-04-14

**Authors:** Giovana de Godoy Fernandes, Bruna Pedrina, Patrícia de Faria Lainetti, Priscila Emiko Kobayashi, Verônica Mollica Govoni, Chiara Palmieri, Veridiana Maria Brianezi Dignani de Moura, Renée Laufer-Amorim, Carlos Eduardo Fonseca-Alves

**Affiliations:** 1 School of Veterinary Medicine and Animal Science, São Paulo State University—UNESP, Botucatu 18618-681, Brazil; giovana.godoy@unesp.br (G.d.G.F.); bruna.pedrina@unesp.br (B.P.); patricia.lainetti@unesp.br (P.d.F.L.); priscila.e.kobayashi@unesp.br (P.E.K.); veronica.m.govoni@unesp.br (V.M.G.); renee.laufer-amorim@unesp.br (R.L.-A.); 2 Gatton Campus, School of Veterinary Science, The University of Queensland, Gatton, QLD 4343, Australia; c.palmieri@uq.edu.au; 3 School of Veterinary Medicine and Animal Science, Federal University of Goiás, Goiânia 18531-883, Brazil; vdmoura@ufg.br; 4 Institute of Health Sciences, Paulista University-UNIP, Bauru 17048-290, Brazil

**Keywords:** dog, comparative oncology, inflammation, prostatic atrophy, preneoplastic lesion

## Abstract

**Simple Summary:**

Prostatic diseases are important worldwide, being the prostate cancer (PC) the most common tumor in men. Among the factors associated with PC development, the preneoplastic lesions are well-recognized. Preneoplastic lesions are cellular morphological alterations, induced by different factors and present a potential to progression for PC. In this scenario, dogs are considered spontaneous models. Dogs naturally develops prostatic hyperplasia, preneoplastic lesions and PC. Among the preneoplastic lesions, the proliferative inflammatory atrophy (PIA) develops spontaneously in dogs. PIA is an epithelial lesion induced by prostatic chronic inflammation, leading to a proliferative atrophy of the prostate gland. Thus, this study aimed to perform a full PIA morphological, phenotypical and molecular characterization in dogs. After reviewing the archives of the veterinary pathology service, it was identified 171 dogs containing PIA in the prostate gland, and among the PC cases (*N* = 84), it was identified PIA lesions surrounding 60.7% of PC cases. Besides that, we identified loss of genes related to the maintenance of prostatic tissue and can predispose to malignant transformation. Moreover, mutations in androgen receptor gene were identified, demonstration alteration in DNA in PIA. Overall, these results support the hypothesis that PIA can be considered a preneoplastic lesion in canine prostate.

**Abstract:**

Proliferative inflammatory atrophy (PIA) is an atrophic lesion of the prostate gland that occurs in men and dogs and is associated with a chronic inflammatory infiltrate. In this study, we retrospectively reviewed canine prostatic samples from intact dogs, identifying 50 normal prostates, 140 cases of prostatic hyperplasia, 171 cases of PIA, 84 with prostate cancer (PC), 14 with prostatic intraepithelial neoplasia (PIN) and 10 with bacterial prostatitis. PIA samples were then selected and classified according to the human classification. The presence of PIA lesions surrounding neoplastic areas was then evaluated to establish a morphological transition from normal to preneoplastic and neoplastic tissue. In addition, the expression of PTEN, P53, MDM2 and nuclear androgen receptor (AR) were analyzed in 20 normal samples and 20 PIA lesions by immunohistochemistry and qPCR. All PIA lesions showed variable degrees of mononuclear cell infiltration around the glands and simple atrophy was the most common histopathological feature. PIA was identified between normal glands and PC in 51 (61%) out of the 84 PC samples. PIA lesions were diffusely positive for molecular weight cytokeratin (HMWC). Decreased PTEN and AR gene and protein expression was found in PIA compared to normal samples. Overall, our results strongly suggest that PIA is a frequent lesion associated with PC. Additionally, this finding corroborates the hypothesis that in dogs, as is the case in humans, PIA is a pre neoplastic lesion that has the potential to progress into PC, indicating an alternative mechanism of prostate cancer development in dogs.

## 1. Introduction

The contribution of inflammation to prostate carcinogenesis is well-known [1,2], occurring through a combination of repeated damage to the genome and increased cell proliferation [3]. The inflammatory process in the prostate gland is associated with a morphologically atrophic epithelium, characterized by a high proliferative index and decreased expression of apoptotic markers [1,3]. De Marzo et al. [4] proposed the term proliferative inflammatory atrophy (PIA) to designate the discrete focus of glandular epithelial proliferation with the morphological appearance of simple atrophy, surrounded by variable degrees of inflammation.

In men, PIA occurs in the peripheral zone of the prostate gland, where prostate cancer (PC) is also more commonly observed [4]. A mononuclear inflammatory infiltrate is frequently associated with PIA lesions [4,5,6] and these inflammatory cells secrete proteases, as well as mitogenic, antiapoptotic and angiogenic factors in the prostatic microenvironment [7], which ultimately induce epithelial cell atrophy, followed by cell proliferation [5,8]. PIA can occur adjacent to high-grade prostatic intraepithelial neoplasia (HGPIN) and prostate cancer (PC), with some studies highlighting the preneoplastic significance of PIA in human PC development [9,10]. The most accepted theory suggests a progression from PIA to HGPIN and subsequently PC [10,11]. De Marzo et al. [4] previously characterized the morphology and immunophenotype of PIA, describing 34% of focal atrophic lesions surrounding high-grade intraepithelial neoplasia (PIN), and thus, hypothesizing a morphological progression from PIA to HGPIN. PIA has been described in the canine prostate [12,13,14,15,16], although only from a morphological perspective, without analyzing its preneoplastic potential.

Chromosomal abnormalities were previously described in canine PIA lesions. Copy number gain was detected in *CRYGS*, *ADIPOQ, SST* genes and copy number losses were identified in *CD38*, *ZNF518B*, *WDR1*, *SLC2A9* genes [16]. These results demonstrated the occurrence of chromosomal instability in canine PIA lesions and represent the first evidence of their premalignant potential. Loss of E-cadherin expression has also been reported in canine PIA [15]. Normally dividing prostatic epithelial cells can lose E-cadherin during cell division and re-express the same protein after the replication is completed [17]. Thus, all these previous studies suggest the occurrence of several genomic and protein alterations in canine PIA, that favors its potential preneoplastic lesion.

This study aimed to evaluate the immunophenotype of PIA lesions in dogs, establish a topographic relationship between normal, PIA and PC lesions, as well as to analyze TP53, MDM2, nuclear androgen receptor (AR) and PTEN protein and gene expression in PIA lesions, compared to normal prostates, in order to better characterize its preneoplastic potential in dogs.

## 2. Results

### 2.1. Animal Demographic Data

Prostate samples were collected from adult intact dogs of different ages and breeds. The group of dogs with normal prostate consisted of animals with a median age of 6 years (4–8 years), overrepresented by mixed breed dogs (30/50). Dogs with prostatic hyperplasia (PH) had a median age of 9 years (6–12 years) and this group was also overrepresented by mixed breed dogs (87/140). Among the pure breeds, the American pit bull terrier (15/140) was the most affected. The median age of dogs diagnosed with PIA was 9 years (7–14 years), with mixed breed dogs being the most affected (95/171). PC-affected dogs had a median age of 11 years (9–16 years) with the highest prevalence in mixed breed dogs (21/84). The median age of dogs with PIN and bacterial prostatitis was 9 (7–10) and 7 (5–8) years, respectively.

### 2.2. Morphology and Immunophenotype of PIA

**Figure 1:**
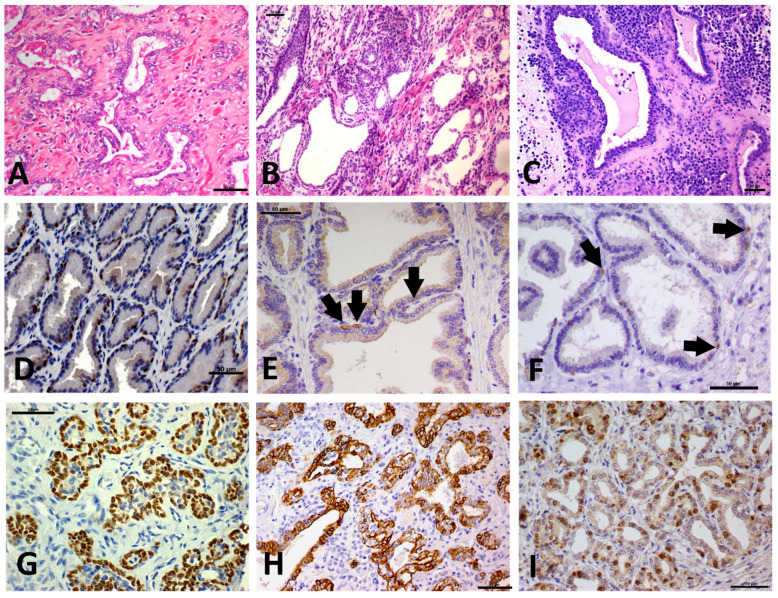
Hematoxylin and eosin (H&E) staining and immunohistochemistry of canine prostate with proliferative inflammatory atrophy (PIA). (**A**): Discrete mononuclear inflammatory infiltrate admixed with multifocal atrophic glands with at least two layers of epithelial cells. (**B**): Moderate mononuclear inflammatory infiltrate and areas of prostatic gland atrophy with cells showing hyperchromatic nuclei and at least two layers of epithelial cells. (**C**): Severe mononuclear inflammatory infiltrate with atrophic epithelial cells showing at least two layers of epithelial cells, hyperchromatic nuclei and evident nucleolus. (**D**): Nuclear p63 expression in a normal prostate gland. There is a discontinuous basal cell layer. (**E**): High molecular wight cytokeratin expression by normal basal cells (arrows). (**F**): Nuclear Ki67 expression by normal basal cells (arrows). There is an absence of Ki67 in luminal epithelial cells and only scatted basal cells express Ki67. (**G**): Diffuse P63 expression by atrophic cells in a PIA. (**H**): Diffuse membranous expression of high molecular weight cytokeratin in a PIA. (**I**): Nuclear positive Ki67 expression by atrophic cells in a PIA lesion. Scale bar = 50 μm.

All PIA lesions (*N* = 171) were surrounded by mononuclear cells, and specifically, by a low inflammatory infiltrate (Figure 1A) in 35.5% of samples (61/171), moderate inflammation in 42.1% of samples (72/171) (Figure 1B) and severe inflammatory infiltrate in 22.4% (38/171) (Figure 1C). Simple atrophy was the most common histopathological feature, observed in 73% of all cases (125/171). A mixed pattern was prevalent in 17.5% of cases (30/171) and post atrophic hyperplasia (PAH) in 9.5% (16/171).

**Figure 2:**
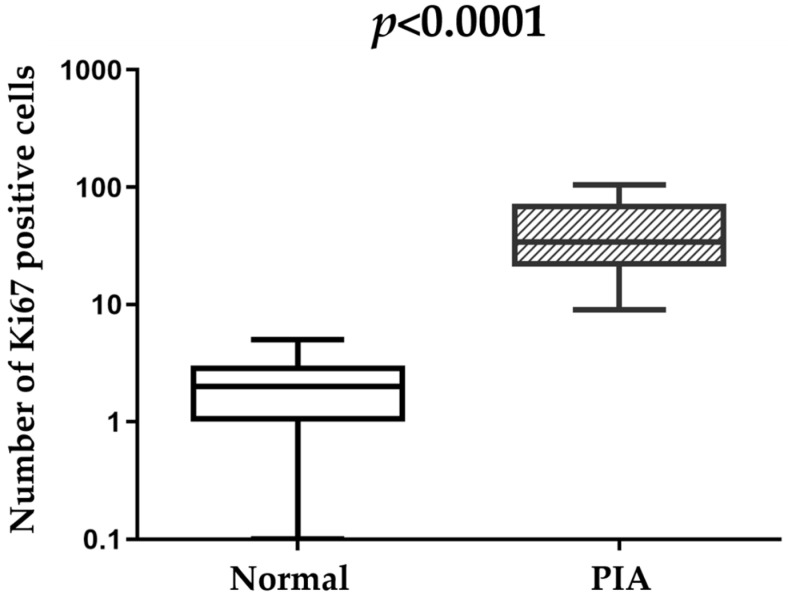
Ki67 protein expression in normal and proliferative inflammatory atrophy (PIA) samples. A high Ki67 index was observed in PIA compared to normal samples, indicating a high proliferative rate in PIA (*p* < 0.0001).

In normal prostatic tissue, high molecular weight cytokeratin (HMWC) (Figure 1D) and P63 (Figure 1E) were expressed by basal cells, showing a discontinuous layer (100%—20/20). Interestingly, PIA lesions showed a continuous basal cell layer that was positive for P63 (Figure 1D) and HMWC (Figure 1E); with a score of four for both markers. The normal prostatic tissue had no Ki-67 expression in the epithelial luminal cells; only a few basal cells were Ki-67 positive (Figure 1F). The normal prostatic tissue had a mean of 2.2 ± 1.5 Ki-67 positive cells, while PIA lesions (Figure 1I) showed a mean of 45.1 ± 31.8 Ki-67 luminal and basal positive cells with a statistically significant difference between the two groups (*p* < 0.0001) Figure 2.

**Figure 3:**
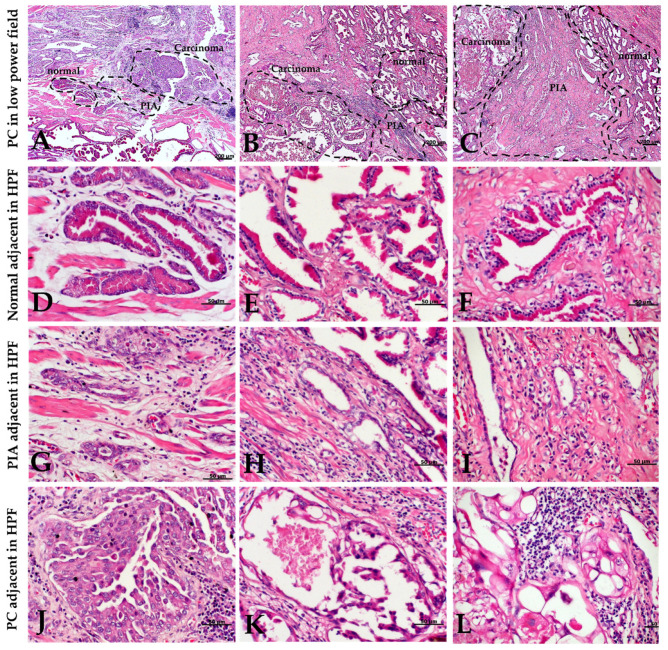
Hematoxylin and eosin (H&E) staining from three different canine prostate carcinomas (PC). (**A**–**C**): Low power field containing a carcinoma area surrounded by PIA and a normal adjacent area. A clear morphological transition between normal, proliferative inflammatory atrophy (PIA) and PC areas is evident. (**D**–**F**): High power field (inset of figures (**A**–**C**), respectively) of adjacent normal tissue. Note the prostatic gland epithelium consisting of columnar and basal epithelial cells. (**G**–**I**): High power field of surrounding PIA (areas outlined by the plotted areas in figures (**A**–**C**)). Characteristic simple atrophy with one epithelial layer and hyperchromatic nuclei. (**J**–**L**): High power field of a carcinoma area. (**J**): Neoplastic cells disposed in nests and moderate pleomorphism. (**K**): Cells present a pseudopapillary shape with presence of degeneration. (**L**): Ballooning neoplastic cells showing degeneration associated intense surrounding inflammation. HPF: High power field.

All samples with PIN (14/14) contained adjacent PIA lesions. No PIN foci were observed adjacent to PC lesions. A total of 61% (51/84) of the PC samples had PIA lesions close to the neoplastic areas and an evident histological transition from normal tissue (normal or hyperplastic) to PIA and PC was observed in 21.5% (11/51) of the PC samples (Figure 3).

### 2.3. Immunohistochemical Features

**Figure 4:**
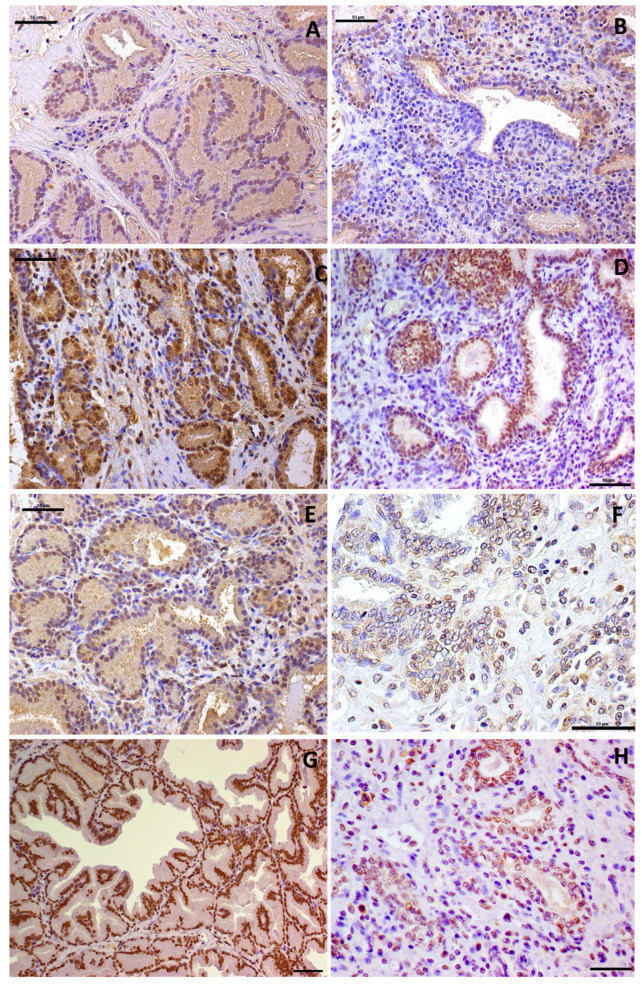
Immunohistochemical staining of the normal canine prostate and canine proliferative inflammatory atrophy (PIA). (**A**): PTEN expression in a canine normal sample: nuclear and cytoplasmic positive staining of luminal epithelial cells. (**B**): PTEN expression in proliferative inflammatory (PIA) samples: atrophic prostatic luminal cells with a lack of PTEN expression. (**C**): Nuclear and cytoplasmic P53 expression in a normal canine prostatic sample and (**D**): PIA lesions. (**E**,**F**): Positive cytoplasmic and nuclear MDM2 expression in normal (**E**) and PIA (**F**) samples. (**G**,**H**): nuclear androgen receptor (AR) expression in a normal prostatic sample and lack of expression in PIA samples (**H**). Counterstaining with Harris hematoxylin, DAB, 20×.

The immunohistochemical results are presented in Table 1. Both basal and luminal cells were p53 positive, with a nuclear and cytoplasmic expression pattern (Figure 4A). The p53 score was four in nine out of 20 (45%) normal prostatic tissues and three in the remaining cases (11/20). PIA samples were assigned a score of four in 40% of cases (8/20), a score of three in 40% (8/20) and a score of two in 20% (4/20) (Figure 4B). There was no statistical difference in P53 scores between normal and PIA samples. PTEN expression was only observed in the nuclei and cytoplasm of luminal cells Figure 4C. All normal samples showed more than 75% of positive cells (score of four). Two PIA samples had a score of four, 25% (5/20) had a score of three, 25% (5/20) had a score of two and 40% (8/20) had a score of one (Figure 4D). We identified decreased staining in PIA samples compared to normal samples (*p* = 0.003). MDM2 was expressed in the nuclei of luminal and basal cells (Figure 4E) of one normal sample (5%) with a score of four, 30% (6/20) of samples with a score of three and 65% (13/20) with a score of two. Fifteen percent (3/20) of PIA samples showed a score of four, 30% (6/20) showed a score of three and 55% (11/20) showed a score of two (Figure 4F). There was no statistical difference in MDM2 expression between normal and PIA samples.

AR was positive in luminal cells and occasionally in basal cells (Figure 4G). All normal prostate samples (20/20) showed more than 75% AR-positive cells. The expression pattern was significantly decreased in PIA samples compared to normal samples (*p* = 0.01). Fifty-five percent (11/20) of PIA samples showed a score of three and 45% (9/20) had a score of two (Figure 4H).

### 2.4. Gene Expression

There was no difference in *TP53* and *MDM2* transcript levels between normal and PIA samples (*p* > 0.05). A positive correlation between *TP53* and *MDM2* transcript levels in normal (R = 0.7754; *p* < 0.0001) and PIA (R = 0.6573; *p* = 0.0202) samples was observed, suggesting that in both cases the increased *TP53* transcript level is correlated with a simultaneous increased expression of *MDM2* (Supplementary Figure S1). No statistical correlations were observed between the remaining comparisons.

**Figure 5:**
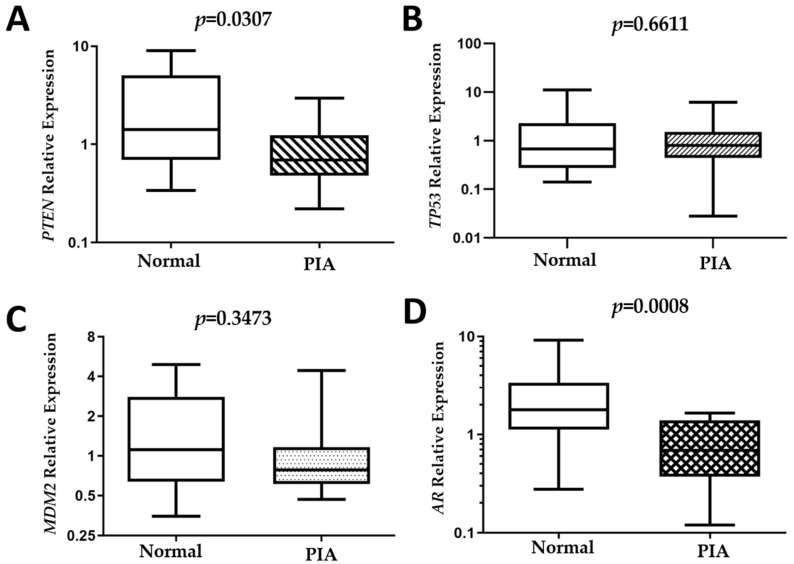
Evaluation of PTEN, TP53, MDM2 and AR gene expression in normal compared to proliferative inflammatory atrophy (PIA) samples. (**A**) Decreased PTEN expression was observed in PIA samples compared to normal samples (**A**). TP53 (**B**) and MDM2 (**C**) transcripts showed no statistically significant differences. AR expression decreased in PIA samples compared to normal samples (**D**).

*PTEN* (*P* = 0.0307) and *AR* (*P* = 0.0008) expression was decreased in PIA compared to normal samples (Figure 5). The median relative quantification (RQ) of *AR* in normal samples was 1.8 (0.3–9), while in PIA samples it was 0.7± 0.1–1.6. The median *PTEN* RQ was 1.4 ± 0.3–6.7 and 0.7 ± 0.2–3 in normal and PIA samples, respectively.

### 2.5. Matrix of Multiple Correlation

**Figure 6:**
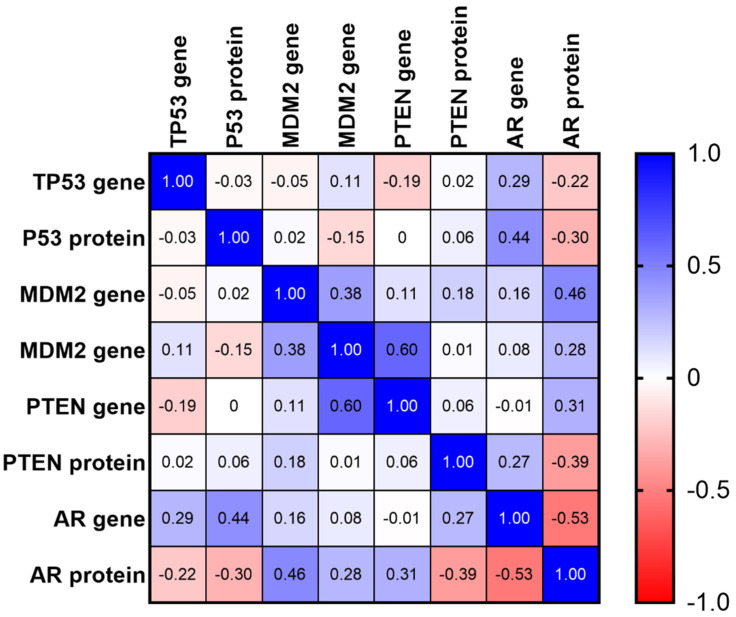
Matrix of multiple correlation among PTEN, TP53, MDM2 and AR gene and protein expression. The red color represents negative correlation and the blue color represents a positive correlation. The color intensity is associated with a strongest correlation. The blue color represents a positive correlation and the red color represents a negative correlation.

Using a matrix of multiple correlation among PTEN, P53, MDM2 and AR genes and proteins, a positive strong correlation was demonstrated between *PTEN* and *MDM2* gene expression (Spearman r = 0.6) and between *AR* and *MDM2* gene expression. The matrix of multiple correlation did not show a positive correlation with the respective gene and protein expression (Figure 6).

### 2.6. AR Sequencing

**Figure 7:**
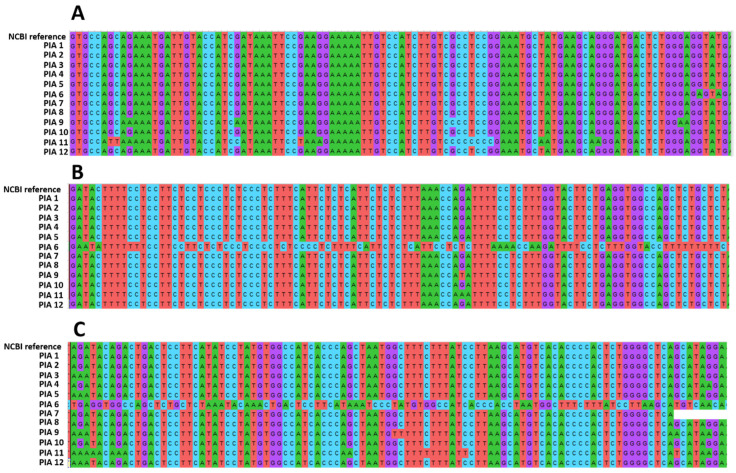
Androgen receptor (AR) sequencing analysis of the 12 proliferative inflammatory atrophy (PIA) samples after alignment. Each figure (**A**–**C**) represents different regions of the AR gene alignment. There are six samples containing mutations, with PIA 6 representing the samples with a higher number of nucleotide modification. (**A**,**B**): sequencing alignment showing mutations in PIA6, PIA9 and PIA 11 samples. C: Sequencing alignment evidencing mutation in the samples PIA3, PIA4, PIA5, PIA6, PIA9, PIA11.

All samples were amplified in PCR analysis in the electrophoresis. However, after sequencing, eight out of 20 samples showed a very low sequencing quality and were excluded from the alignment analysis. In the PIA samples aligned in the investigation of *AR* mutations (*N* = 12), six out of 12 samples (50%) showed mutations, with one sample showing several nucleotide alterations (Figure 7).

## 3. Discussion

In human PC, the progression of PIA to high grade-PIN (HGPIN) and PC cancer has been described over recent years, due the role of chronic inflammation in cancer development [1,2,3,4,5,6]. In dogs, little is known in regard to the carcinogenic process and the progression of preneoplastic lesions to PC. It seems that canine PC develops from androgen-independent cells and most cases of canine PC have been found to be negative for AR [16]. Thus, alterations to the AR receptor are considered to be one of the most important steps in PC development. The previously published literature has focused on studying PIA as a preneoplastic lesion and has shown copy number variations, indicating molecular instability [16]. Although an increased number of studies have been published in recent years, the carcinogenic process of canine PC development is still unclear.

Based on this gap in the literature, our results suggest that PIA is a very common histological lesion in the prostates of intact dogs, with a prevalence of 36.5% in our dataset. On the other side, PIN was present in only 3% of selected cases (14/469). The real incidence of PIN lesions in the canine prostate is controversial. Waters and Bostwick [18,19] reported an incidence of 55–65%, while in two larger studies conducted by Aquilina et al. [20] and Madewell et al. [21], the incidence was low (less than 3%) without any histological association between PIN and PC [20,21]. Thus, PIN lesions have been most likely overestimated or not correctly diagnosed on histological sections and their role in canine prostatic carcinogenesis is still unknown.

The studied population was based on intact dogs, because in Brazil, castration of male dogs is not routinely performed due to cultural factors. Interestingly, we did not see the frequent occurrence of prostate cancer in castrated dogs. This is important in studies evaluating PIA, because castrated dogs show atrophy of the prostate during hormone deprivation and present hormonal atrophy instead of an inflammatory atypical atrophy.

PIA is a common finding in the canine prostate, although its pathogenesis, mechanisms of occurrence and role on prostatic carcinogenesis are unclear. PIA lesions surrounded canine PC areas in 61% of carcinoma samples and, in 21.5% of these cases, the morphological transition between benign tissue, PIA lesions and invasive prostate cancer was evident. Taking into account previously published articles assessing the molecular basis of canine PIA, in addition to the findings from the present study, this result may suggest a potential association between PIA and PC. The morphological transition term was previously introduced by Wang et al. [22] to associate PIA as a preneoplastic lesion in human prostatic pathology. Morphological transition refers to normal, followed by PIA areas colliding to prostatic carcinoma in histological specimens. In our samples, PIA lesions were characterized by a higher proliferative index (shown by Ki67) compared to normal prostates, underlining their high proliferative potential. Previously, our research group investigated the proliferative index of 12 PIA and 18 PC samples, demonstrating a median Ki67 expression level of 54.5 and 366, respectively [23]. Ki67 expression is higher in PIA and PC compared to normal samples. In the carcinogenic process of the human prostate, a dynamic progression from low-PIN, to HGPIN, and finally PC is widely accepted [5,24]. De Marzo et al. [4] then identified PIA lesions as a new precursor of HGPIN. Thus, the carcinogenic process of human and canine prostates might occur through different, a yet unexplored, mechanisms.

Canine PC is characterized by a heterogeneous pattern of cytokeratin expression, as attested by the consensus reached on the intermediate phenotype (luminal markers+ /basal markers+) of canine PC [12,13,25,26,27]. As evidenced in this study, intermediate cells are also found in greater quantities in PIA lesions, similar to the prevalent cell population of PIA in humans. It is likely that, even in dogs, PIA lesions arise from the proliferation of basal cells stimulated by the inflammatory environment. During normal prostate development and differentiation, basal cells proliferate, reducing the expression of basal cells markers (CK5, HMWC and P63) and overexpressing luminal markers (CK18), until they completely lack the basal cell markers and express luminal markers only [28]. Due to the high proliferative index of PIA lesions, demonstrated by the expression of Ki67, it is most likely that cells are constantly stimulated to divide without further developing into luminal cells. Interestingly, many canine PCs show an intermediate phenotype (concomitant expression of P63 or CK5 or CK8/18) [13,27,29]. Thus, the same cell phenotype may be involved in the development of both PIA and PC in dogs and both lesions may have a common origin in most—but not all—cases.

PTEN, P53, MDM2 and AR dysregulation in human prostate cancer has been widely described [30,31,32]. PTEN and P53 downregulation plays an important role in cell growth and apoptosis [33]. Furthermore, *TP53* and *MDM2* genes play a dual role during cell proliferation: *MDM2* is an oncogene associated with the proliferation of prostatic cells and *TP53* acts as an *MDM2* inhibitor [30]. During PC progression, tumor cells become androgen-independent and the AR inhibition of human prostate cells promotes cell migration and invasiveness [34].

P53 and MDM2 protein expression were similar in normal prostates and PIA, although a positive correlation between *TP53* and *MDM2* transcript levels in both cases was observed, i.e., higher *TP53* transcripts had higher *MDM2* levels. This correlation could suggest a role of *MDM2* in controlling *TP53* transcripts, as is the case in the human prostate [35]. *TP53* and *MDM2* expression have been widely studied in human cancers and *MDM2* is a negative regulator of the *TP53* transcript [35,36,37]. In canine PC, upregulation of *MDM2* and downregulation of *TP53* transcript levels have previously been described [38]. Recently, *TP53* copy number loss and *MDM2* gains were described in canine PC, suggesting that P53 and MDM2 could be important drivers in canine prostatic carcinogenesis [16]. Since P53 and MDM2 were not deregulated in PIA samples, and both markers have instead been described as altered in PC [16,38], P53 downregulation and MDM2 overexpression may occur only in the advanced stage of prostatic carcinogenesis, after malignant transformation.

Our results show downregulation of the AR gene and protein levels in PIA samples, as compared to normal prostate samples. The *AR* gene has a key role in prostatic development and maintenance [39]. In dogs, downregulation of the *AR* gene is common in PC development [39]. Previously, our research group demonstrated an *AR* copy number loss in canine PC [16], indicating *AR* loss as an important event in canine PC development. Since we demonstrated the downregulation of *AR*, this gene may be altered in the early stages of neoplastic development and this result supports the hypothesis of the potential progression from PIA to PC. The lack of *AR* expression in canine PC has been widely demonstrated in previous studies [16,25,26,27,38]. Additionally, our results have demonstrated that PIA with *AR* downregulation may be a precursor lesion to PC. Together, this information reinforces the idea of PIA progression into invasive PC in dogs. However, since we demonstrated an intermediate phenotype for PIA lesions, the AR downregulation may occur due the cell differentiation from basal cells into luminal cells. Thus, this result should be evaluated carefully.

Another gene undergoing downregulation in PIA lesions is *PTEN*. In human PC, PTEN loss is correlated with PC androgen independence [40]. PTEN downregulation is associated with decreased AR levels and these alterations are associated with activation of the anti-apoptotic pathway [40,41]. In knockout *PTEN* mice (Pten+/−), used as models of prostate carcinogenesis, the animals developed spontaneous inflammation with a high number of preneoplastic lesions and carcinomas compared to the controls [42]. Thus, *PTEN* loss is likely important during the carcinogenic process of the prostate gland [42].

Mice are the classical animal models used for studying the development of PIN and PC [43]. However, even though they may experience high-grade inflammation, PIA cannot be induced in these experimental models [42]. Thus, dogs represent a unique opportunity for comparative studies in order to evaluate the role of PIA as preneoplastic lesions in the prostate.

The high proliferative index and the downregulation of *PTEN* and *AR* in PIA lesions may reflect their preneoplastic potential. The PIA premalignant potential is strongly supported by the high mutation index identified in our sequencing analysis. A total of six out of 12 samples (50%) showed mutations, and canine PC is strongly associated with AR decreased expression (even in castrated dogs). Thus, mutations in the AR gene can lead to the progression of PIA to PC in intact dogs.

## 4. Materials and Methods

### 4.1. Animals and Experimental Design

This study was performed in accordance with the National and International Recommendations for the Care and Use of Animals (National Research Council) [44]. All procedures have been approved by the Ethics Committee on Animal Use (CEUA) of the Veterinary Teaching Hospital of São Paulo State University (CEUA/UNESP, #0208/2016). All samples were obtained from Brazilian dogs.

### 4.2. Tissue Selection

Four hundred and sixty nine hematoxylin and eosin (H&E) slides from different canine prostates were selected from the Veterinary Pathology archive of São Paulo State University—UNESP, between 2011 and 2019. Histological evaluation of normal, PIN, and PC samples was performed according to the human WHO Tumors of the Urinary System and Male Genital Organs guidelines [45], which were recently adapted to canine PC [14]. PIA lesions were identified based on morphological features, as described by De Marzo et al. [4]. All samples were from adult intact dogs: 50 normal prostates, 140 prostatic hyperplasia, 171 proliferative inflammatory atrophy (PIA), 84 prostate carcinoma (PC), 14 prostatic intraepithelial neoplasia (PIN) and 10 bacterial prostatitis samples were obtained. The H&E slides from the 84 PC cases were selected to evaluate PIA and PIN surrounding the neoplastic tissue, as previously described [22].

### 4.3. PIA Morphological Features

The intensity of inflammation was scored according to De Marzo et al. [4] with modifications. Briefly, all H&E PIA slides (*N* = 171) were evaluated using a numerical scale from 0 to 6, with 0 representing no inflammation, 1 and 2 mild inflammation, 3 and 4 moderate inflammation and 5 and 6 severe inflammation. The key morphological findings to identify PIA lesions in a low power field (5×) were overall hyperchromatic appearance, associated inflammatory infiltrate, loss of papillary architecture and cuboidal cell morphology. In a higher power field, key findings were acini showing at least two layers of epithelial cell, atrophic appearance of cuboidal cells with scant cytoplasm, and in some cases, the presence of evident nucleolus and mitotic figures [4,12].

Lesions were divided into simple atrophy, PAH and mixed pattern (simple atrophy and PAH), according to De Marzo et al. [4]. The presence of dilated glands was also evaluated and referred to as cystic atrophy.

### 4.4. Immunohistochemistry

Twenty normal prostate samples and 20 PIA paraffin blocks were further selected to investigate both gene and protein expression of specific markers as outlined below. Immunohistochemistry for HMWC, P63, Ki67, PTEN, P53, MDM2 and androgen receptor (AR) was performed with antibodies that had been previously validated to cross-react with canine tissue samples [15,38]. Slide sections were dewaxed in xylol and rehydrated in graded ethanol. For antigen retrieval, the slides were incubated with citrate buffer (pH 6.0) in a pressure cooker (Pascal^®^; Dako, Carpinteria, CA, USA). The slides were treated with freshly prepared 3% hydrogen peroxide in methanol for 20 min and further washed in Tris-buffered saline. The primary antibodies were diluted to 1:500 for PTEN (Bioss, Massachusets, USA -Bs-0686R), 1:50 for MDM2 (Abcam, Cambridge, UK-ab38618), 1:50 for P53 (Santa Cruz Biotecnology, sc-75366), 1:100 for AR (Abcam, Cambridge, UK-ab77557), 1:300 for HMWC (DakoCytomation, clone: 34βE12), 1:100 for P63 (DakoCytomation, clone 4A4), 1:300 for Pan-cytokeratin (Invitrogen), 1:50 for Ki-67 (DakoCytomation, clone: MIB1) with an overnight incubation at 4 °C. A polymer system (Envision, Dako, Carpinteria, CA, USA) was applied as a secondary antibody conjugated to peroxidase and 3′-diaminobenzidine tetrahydrochloride (DAB1, Dako, Carpinteria, CA, USA) was used as the chromogen, for 5 min, followed by Harris hematoxylin counterstain.

Negative controls were performed for all antibodies by omitting the primary antibody, replacing them with Tris-buffered saline and also with an iso-type matched immunoglobulin according to Hewitt et al. [46]. Normal prostate was used as a positive control for AR, p63, HMWC and PTEN. A normal lymph node was used as a positive control for p53, MDM2 and Ki67 according to the Human Protein Atlas recommendation (www.proteinatlas.org, accessed on 15 November 2020).

### 4.5. Immunohistochemical Score

As regards P53, MDM2, AR, PTEN, P63 and HMWC, samples were scored based on the percentage of positive cells for each antibody with the following scores: 1–10% positive cells (score 0), 11–25% positive cells (score 1); 26–50% positive cells (score 2); 51–75% positive cells (score 3); and more than 76% positive cells (score 4). The immunohistochemical results were independently interpreted by three investigators (C.E.F.A., P.E.K. and R.L.A.). The distribution (basal or luminal cells) of P63 and HMWC was also evaluated. Ten high power fields were used to establish the final IHC score. Ki-67 analysis was made by counting the number of positive cells per total of 1000 cells.

### 4.6. Laser-Capture Microdissection and qPCR

For mRNA extraction, microdissection was performed using a Leica AS LMD laser-capture system (Leica Microsystems, Wetslzar, Germany), according to [47] with modifications. mRNA analysis was performed with 20 normal and 20 PIA fresh frozen tissues (same animal prostates as those used for immunohistochemistry). Briefly, 10 µm tissue sections were cut in a cryostat (Leica Microsystems) and mounted on Nuclease and Human Nucleic acid free PEN-membrane slides 2.0 µm (MembraneSlides, Leica Microsystems). The slides were stained (H&E) and once air-dried, PIA areas were microdissected [47]. mRNA was extracted with RNeasy Micro Kit (Qiagen, Hilden. Germany) according to the manufacturer instructions. cDNA was synthesized in a final volume of 20 µL, and each reaction contained 1 µg of total RNA treated with DNAse I (Life Technologies, Rockville, MD, USA), 200 U of SuperScript III reverse transcriptase (Life Technologies), 4 µL of 5X SuperScript First-Strand Buffer, 1 µL of each dNTP at 10 mM (Life Technologies), 1 µL of Oligo-(dT)18 (500 ng/µL) (Life Technologies), 1 µL of random hexamers (100 ng/µL) (Life Technologies), and 1 µL of 0.1 M DTT (Life Technologies). Reverse transcription was performed for 60 min at 50 °C, and the enzyme was subsequently inactivated for 15 min at 70 °C. cDNA was stored at −80 °C.

*TP53, MDM2, AR* and *PTEN* and the endogenous genes (Table 2) were conducted in a total volume of 10 μL containing Power SYBR Green PCR Master Mix (Applied Biosystems; Foster City, CA, USA), 1 μL of cDNA (1:10) and 0.3 μM of each primer. The reactions were performed in triplicate in 384-well plates using QuantStudio 12K Flex Thermal Cycler equipment (Applied Biosystems; Foster City, CA, USA). A dissociation curve was included in all experiments to determine the PCR product specificity. Relative gene expression was quantified using the 2-ΔΔCT method [48].

### 4.7. DNA Extraction and Sequencing

DNA extraction of 20 PIA tissues used in mRNA expression was performed using DNeasy Blood and Tissue Kits (Ambion, Life Technologies, MA, USA), according to manufacturer’s instructions. The amplification of the androgen receptor (AR) DNA-binding domain was performed in a Veriti 96-Well Thermal Cycler (Thermo Fisher Scientific, MA, USA). The methodology was performed according to the previously described by Rivera-Calderon et al. [49]. Briefly, 5 μL of 10× PfuUltra II, 0.5 μL of 100 μM dNTP, 1 μL of each oligonucleotide in 10 μM of each primer, 1 μL of genomic DNA (100 ng/μL) and 1 μL PfuUltra II fusion HS DNA polymerase (Agilent, CA, USA). The primers used were previously published by Lai et al. [50].

After of the amplification, the AR DNA-binding domain was analyzed in 2% agarose gel stained with Neotaq Brilliant Plus DNA Stain (Neobio, Brazil). Then, the PCR products were cut from the agarose gel were and the DNA sequencing was evaluated with a BigDye™ Terminator v 3.1 Cycle Sequencing Kit version 3.1, according to the manufacturer’s instructions (Applied Biosystems, Thermo Fisher Scientific, MA, USA). The RT-amplicons were directly sequenced at ABI 3500 (Applied Biosystems, Thermo Fisher Scientific, MA, USA). The resulting nucleotide sequences were compared to data of the NBCI (National Center for Biotechnology Information, Bethesda, MD, USA) (gi:6578766).

Sequences analysis was performed in the MEGA Software (www.megasoftware.net, accessed on 17 February 2021) [51], a FASTA analysis was performed, and the sequences aligned with the reference gene from NCBI (gi:6578766), according to the previous literature description [50]. Then, sequences with low quality were excluded.

### 4.8. Data Analysis

Statistical analyses were performed using GraphPad Prism v.5.0 (GraphPad Software Inc., La Jolla, CA, USA). Kruskal–Wallis or Mann–Whitney U tests was applied to compare TP53, MDM2, AR and PTEN transcription levels between normal and PIA samples. The chi-square exact test was used to evaluate difference in the immunohistochemical expression between normal and PIA samples.

### 4.9. Data Availability

The authors confirm that the data supporting the findings of this study are available within the article and/or its Supplementary Materials.

## 5. Conclusions

Canine PIA is a common lesion in the dog prostate and shares many morphological similarities with human PIA. Our results strongly suggest that PIA is a potential pre neoplastic lesion and is associated with the progression to PC.

## Figures and Tables

**Table 1:** PTEN, P53, MDM2 and AR immunohistochemical expression in canine normal prostate and PIA samples.

		Score				
	Group	1	2	3	4	*p*
PTEN	Normal	0% (0/20)	0% (0/20)	0% (0/20)	100% (20/20)	*p* = 0.003
	PIA	40% (8/20)	25% (5/20)	25% (5/20)	10% (2/20)	
P53	Normal	0% (0/20)	0% (0/20)	55% (11/20)	45% (9/20)	*p* = 0.3568
	PIA	0% (0/20)	20% (4/20)	40% (8/20)	40% (8/20)	
MDM2	Normal	0% (0/20)	65% (13/20)	30% (6/20)	5% (1/20)	*p* = 0.5784
	PIA	0% (0/20)	55% (11/20)	30% (6/20)	15% (3/20)	
AR	Normal	0% (0/20)	0% (0/20)	0% (0/20)	100% (20/20)	*p* = 0.01
	PIA	0% (0/20)	45% (9/20)	55% (11/20)	0% (0/20)	

Legend: Score 1 = 11–25% positive cells; score 2 = 26–50% positive cells; score 3 = 51–75% positive cells; score 4 = more than 75% positive cells.

**Table 2:** Forward and reverse primer sequence for each gene used in RT-qPCR analyses.

Gene Symbol	Location	Primer Sequences
*AR*	Chromosome 24	F: 5′-CGCCCCTGACCTGGTTT-3′
		R: 5′-GGCTGTACATCCGGGACTTG-3′
*PTEN*	Chromosome 26	F: 5′-CGACGGGAAGACAAGTTCATG-3′
		R: 5′-TCACCGCACACAGGCAAT-3′
*MDM2*	Chromosome 10	F: 5′-GGGCCCCTTCGTGAGAATTG-3′
		R: 5′-GGTGTGGCTTTTCTCAGGGATT-3′
*TP53*	Chromosome 5	F: 5′-GAACGCTGCTCTGACAGTAGTGA-3′
		R: 5′-CCCGCAAATTTCCTTCCA-3′
*HPRT*	Chromosome X	F: 5′-AGCTTGCTGGTGAAAAGGAC-3′
		R: 5′-TTATAGTCAAGGGCATATCC-3′

F: forward; R: reverse.

## Data Availability

The data presented in this study are available in Supplementary Materials.

## Supplementary Materials

Supplementary materials can be found at https://www.mdpi.com/article/10.3390/cancers13081887/s1. Figure S1. Canine prostate gland with atrophic lesion. The slides were stained with hematoxylin and eosin staining and tissue area with inflammatory atrophic gland were selected (A). Then, lase microdissection was performed to obtain only the epithelial portion of these atrophic glands (B). Hematoxylin and eosin staining, 200×.
