# Supplementary material for: Morphological and Molecular Characterization of Proliferative Inflammatory Atrophy in Canine Prostatic Samples
Source: Cancers (Basel). 2021 Apr 14;13(8):1887. doi: 10.3390/cancers13081887 (PMC8071022; doi:10.3390/cancers13081887)
Supplement: Supplementary file 1 [file cancers-13-01887-s001.pdf]

cancers MDPI

Supplementary Material: Morphological and Molecular Characterization of Proliferative Inflammatory Atrophy in Canine Prostatic Samples

Giovana de Godoy Fernandes, Bruna Pedrina, Patrícia de Faria Lainetti, Priscila Emiko Kobayashi, Verônica Mollica Govoni, Chiara Palmieri, Veridiana Maria Brianezi Dignani de Moura, Renée Laufer-Amorim and Carlos Eduardo Fonseca-Alves

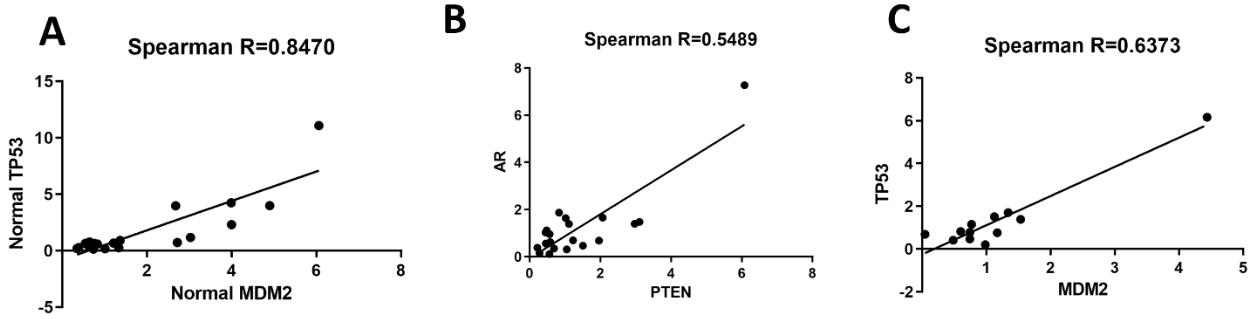

Supplementary Figure S1. Spearman correlation between normal and PIA samples. (**A**)**:** There as a positive correlation between *TP53* and *MDM2* genes in normal samples (Spearman r = 0.847). (**B** and **C**)**:** In PIA samples, it was possible to observe a positive correlation between *AR* and *PTEN* transcripts (Spearman r = 0.5489) and a positive correlation between *TP53* and *MDM2* was also found (Spearman r = 0.6373).
